# Areaalzheimer: development of a digital platform for caregivers based on the results of a needs analysis and mixed-methods pilot evaluation process

**DOI:** 10.3389/fdgth.2025.1730903

**Published:** 2026-01-20

**Authors:** Desiree Piromalli, María Aurora Cañadas-Romero, Marta Ivirico-Prats, Marc Suárez-Calvet, Ana Beriain Bañares, María Sánchez-Valle, Laia Ortíz-Castelví

**Affiliations:** 1 Universitat Abat Oliba CEU, CEU International Doctoral School (CEINDO), Social Research Area, Fundación Pasqual Maragall, Barcelona, Spain; 2 Social Research Area, Fundación Pasqual Maragall, Barcelona, Spain; 3 BarcelonaBeta Brain Research Center (BBRC), Barcelona, Spain; 4 Neurology Department, Hospital del Mar Research Institute, Barcelona, Spain; 5 Department of Communication, Education and Humanities, Universitat Abat Oliba CEU, CEU Universities, Barcelona, Spain; 6 Department of Audiovisual Communication and Advertising, Universidad CEU San Pablo, CEU Universities, Madrid, Spain

**Keywords:** Alzheimer's disease, caregivers, digital platform, participatory development, user-centred design

## Abstract

**Introduction:**

This study describes the user-centred design and evaluation of *AreAreaAlzheimer,* a web-based digital platform developed to support family caregivers of individuals with dementia, especially Alzheimer disease. The initiative sought to ensure that technological solutions effectively address caregivers' actual needs through active user participation at every stage of development.

**Methods:**

Following an iterative, participatory design approach, 419 individuals contributed to the project. The first phase combined a survey of 210 caregivers and focus groups with 22 participants to identify priority support dimensions. Thematic analysis highlighted four main areas: informational guidance, logistical assistance, emotional and communication strategies, and peer social connection. Based on these insights, 147 additional participants provided feedback that refined platform features and content. Finally, platform evaluation included standardized usability measures including the Single Ease Question (SEQ) for task difficulty, the System Usability Scale (SUS) for overall usability perception, the Perceived Usefulness Scale (PUS) completed by 40 caregivers, and scenario-based testing with 19 users who discussed experiences and improvement opportunities.

**Results:**

Quantitative findings showed high ratings in accessibility (average score: 4.5/5), usability (scored 74.3/100), and perceived usefulness was rated lower (average score: 3.4/5). Qualitative feedback supported these results, emphasizing the platform's practical value in everyday caregiving. However, participants with lower digital literacy reported persistent challenges, indicating the need for simplified navigation and adaptive interface features.

**Discussion:**

*AreAlzheimer* demonstrates the potential of participatory design to create inclusive, effective digital health tools for dementia care. Involving caregivers and people living with dementia enriched the design, promoting autonomy and cognitive sensitivity. Future research will integrate these insights into formal scientific protocols to expand participatory digital health innovations in dementia support.

## 1 Introduction

The increase in life expectancy in recent decades has catalysed a significant increase in the elderly population worldwide, which in turn has contributed to a marked increase in the prevalence of age-related neurodegenerative diseases, with Alzheimer's disease (AD) being the most prevalent form of dementia (1). AD accounts for 60%–70% of dementia cases worldwide, and is characterised by progressive cognitive decline, behavioural changes, and loss of autonomy (2). It is estimated that 55 million people worldwide currently live with dementia, a figure expected to almost triple to 152 million by 2050, highlighting a critical public health challenge (1). Spain reflects this global trend, with over 900,000 people currently affected by Alzheimer's disease (AD) or related dementias. This figure is expected to double within the next two decades (3). In Catalonia specifically, around 90,000 people currently live with AD or related dementias (4).

Informal care plays a key role in managing the multifaceted needs of people with dementia, especially as the disease progresses. Informal caregivers, often close family members, but also neighbours or friends, provide unpaid daily support ranging from assistance with activities of daily living, symptom management, and emotional support to coordinating medical care (5–7). Evidently, these caregiving responsibilities entail complex and changing physical and cognitive demands, requiring caregivers to possess specific knowledge and skills to perform their roles effectively (8, 44). Different studies have shown that caregivers, particularly those of people with dementia, experience higher levels of psychological burden and distress compared to caregivers of other populations (9). Emotional stress can lead to increased anxiety and depression and a significant reduction in quality of life, demonstrating the significant impact that caring for others can have on an individual's well-being (10, 11). These emotional challenges are often exacerbated by the practical stresses of managing caregiving tasks, which can lead to fatigue and social isolation (12). Furthermore, studies such as that by Stall et al. (13) have demonstrated that caregiver distress exacerbates the health of individuals with dementia, leading to higher rates of institutionalisation and intensifying the behavioural and psychological symptoms of the disease.

In this context, a wide range of formal interventions have been developed to support these informal caregivers, ranging from educational programmes, psychosocial support and respite care to structured training modules (14–16). These programmes have been shown to reduce caregiver stress, improve quality of life for both caregivers and patients, and reduce healthcare costs, while alleviating the burden on health and social services. However, substantial differences remain between the availability of support interventions and their use and acceptability among caregivers (17).

Contributing factors include rigid service delivery models, a lack of services that meet caregivers' needs, difficulties accessing services, and a failure to consider caregivers' individual circumstances and preferences. Research by Morrisby et al. (18) revealed that services focusing on empowering disabled people and their caregivers achieved better outcomes than unresponsive, inflexible services. According to these authors, caregivers often find it difficult to recognise their own needs, which can hinder access to adequate support. Regarding caregivers of people with AD, the main unmet needs include acquiring specific and in-depth knowledge about AD, receiving guidance on available services and support, managing physical and psychological health, and maintaining social support networks, which are vital for emotional resilience (7, 12).

A particularly neglected area is that of caregivers' information needs. Rutkowski et al. (19) identified a mismatch between information products and caregivers' information behaviour—the generation, acquisition, management, use, communication, and search for information. As Sbaffi et al. (20) state, caregivers require more than just the knowledge that information is available 'somewhere', as each situation is different in terms of the stage of the disease, the severity of the condition, and the geographical location of the support network. Therefore, information must be personalised and context-sensitive rather than generic.

Thus, technology-based support interventions have attracted growing interest as promising solutions to these challenges (17). Digital platforms, mobile health applications, and online educational resources can provide caregivers with timely information, peer support, and training in a scalable, flexible, and personalised way, regardless of geographical and time constraints. For instance, mobile health applications have been shown to improve communication and daily care activities for caregivers of individuals with mild cognitive and communicative impairments (21). However, for digital interventions to be truly effective, they must be carefully tailored to the diverse needs, preferences, and digital literacy levels of caregivers.

Digital literacy is defined here as the ability to use digital technologies effectively and is a determining factor in the acceptance and sustained use of technology among caregivers, many of whom are older adults with limited prior exposure to technology (22). Socioeconomic factors, age, education, and confidence influence digital participation, constituting critical barriers to technology access among this population (23). In Catalonia, the digital divide is particularly pronounced among individuals over 65 years of age (23). The same authors define the digital divide as significant disparities between those who can access and benefit from digitalisation and those who cannot be due to economic, technical, or digital literacy constraints.

Many existing digital platforms are significantly limited by a lack of user-centred design and empirical evaluation that considers the end user's experience (7, 12). Participatory design approaches, which actively involve caregivers throughout the design, development, and evaluation processes, have been identified as the best way to ensure the tools are relevant, usable, and adopted (21, 24). This inclusive methodology facilitates the creation of tools that better address the complex realities of caregiving and the adaptive nature of dementia progression. Rigorous mixed-methods research approaches are essential for properly addressing caregivers' needs and ensuring the quality of technological interaction (25–28).

In contemporary digital health innovation, active user involvement is crucial for developing technologies that effectively support family caregivers of people living with dementia. Co-creation is a collaborative process that brings together diverse stakeholders—including users, clinicians, technical experts and organisational actors—to jointly identify problems, generate ideas and shape solutions. This innovation-driven approach emphasises inclusive participation, shared decision-making and iterative prototyping to produce scalable, sustainable and collectively owned outcomes (29–31). Co-creation fosters dialogue across social and professional boundaries, helping to address structural barriers and enhance health equity by integrating lived user experiences and multiple forms of expertise (29, 31, 32).

As research projects advance from conceptualisation toward development and evaluation, Participatory Design (PD) often becomes the preferred methodology to deepen user engagement. Unlike broader co-creation frameworks, PD centres on the experiential knowledge of end-users, such as family caregivers, involving them continuously through cycles of ideation, prototyping and testing. This dialogic, context-sensitive approach addresses users' real-life needs, cultural considerations and ethical demands, enhancing the usability and acceptability of digital health interventions (30, 31). PD not only ensures that users' voices decisively shape the iterative refinement of solutions but also supports mutual learning between developers and participants, resulting in more adaptable and sustainable technologies (29, 31).

The evolution of digital platforms for family caregivers of people with Alzheimer's disease reveals a continuously transforming field, profoundly shaped by technological innovation and a growing recognition of caregivers' emotional, informational, and organizational needs. Early digital solutions in the 2000s focused primarily on information provision through basic web pages and community forums, marking the initial democratization of specialized knowledge and fostering virtual support communities for geographically or socially isolated caregivers. Over time, digital caregiving tools have advanced to integrate sophisticated technologies—such as artificial intelligence, geolocation, and home automation—which now underpin interactive, personalized systems aimed at improving both care management and caregiver well-being. Current digital platforms for caregivers act as hybrid spaces for technological and social intervention. Their main functions include digital intermediation (linking families with professionals and services), integrated care management (planning, communication, and monitoring), and community-based training and support, thereby empowering caregivers and facilitating informed decision-making. In recent years, the detected need is to move from unidirectional information dissemination to the participatory co-creation of user-centred environments, integrating caregivers' perspectives to address practical, emotional, and relational challenges associated with Alzheimer's care with more impact.

In this context, the project “*Creation of a comprehensive digital platform in the field of Alzheimer's disease in Catalonia”* aims to develop and implement a free, comprehensive, user-centred and scalable digital platform designed specifically to support informal carers of people with AD in Spain and Catalonia. The AreaAlzheimer platform seeks to address unmet informational and training needs and other critical challenges faced by carers through technological empowerment., with the goal of improving well-being, care quality and social inclusion. It embodies a holistic approach to dementia-care support, integrating training, knowledge dissemination, community building and research functions to offer flexible and personalised assistance that responds to users' real-life situations, while also helping to reduce digital inequalities and foster a broad community of caregivers.

The aim of this article is to present the AreaAlzheimer digital platform and the empirical work underpinning its development. More specifically, this study has three research objectives: (1) to identify and analyse the social, informational and technological needs, expectations and practices of family caregivers of people with AD in Catalonia; (2) to translate these findings into the co-design and development of the AreaAlzheimer platform, defining its content, structure and core functionalities across its information, training, community and research components; and (3) to implement and pilot the prototype with family caregivers, assessing its usability, accessibility, perceived usefulness and intention to use.

## 2 Areaalzheimer overview

AreaAlzheimer is part of a project entitled Creation of a Comprehensive Digital Platform in the Field of Alzheimer's Disease in Catalonia, funded by resources granted by the Department of Social Rights within the framework of the Recovery, Transformation and Resilience Plan, which in turn is financed by the European Union through the Next Generation program.

AreaAlzheimer is a bilingual Spanish and Catalan open-access platform aiming to support informal caregivers of people with Alzheimer's disease.

**Figure 1:**
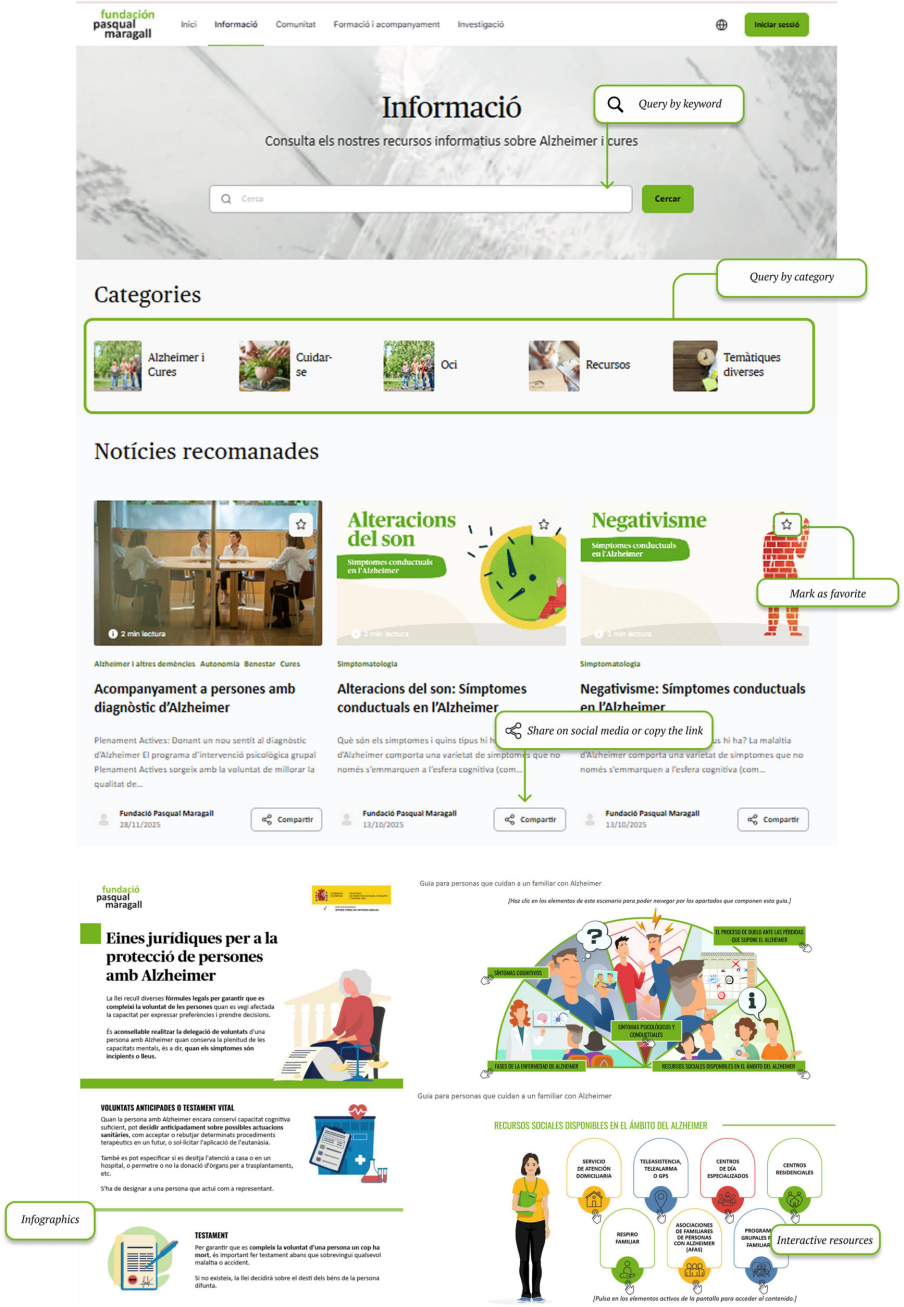
General overview of the information section. Screenshot from: Pasquall Maragall Foundation, https://areaalzheimer.fpmaragall.org/.

The information section categorises Alzheimer's resources and topics based on their most preferred content. Content is tagged to connect it with user preferences. This way, all users have access to a personalized content section (Figure 1).

**Figure 2:**
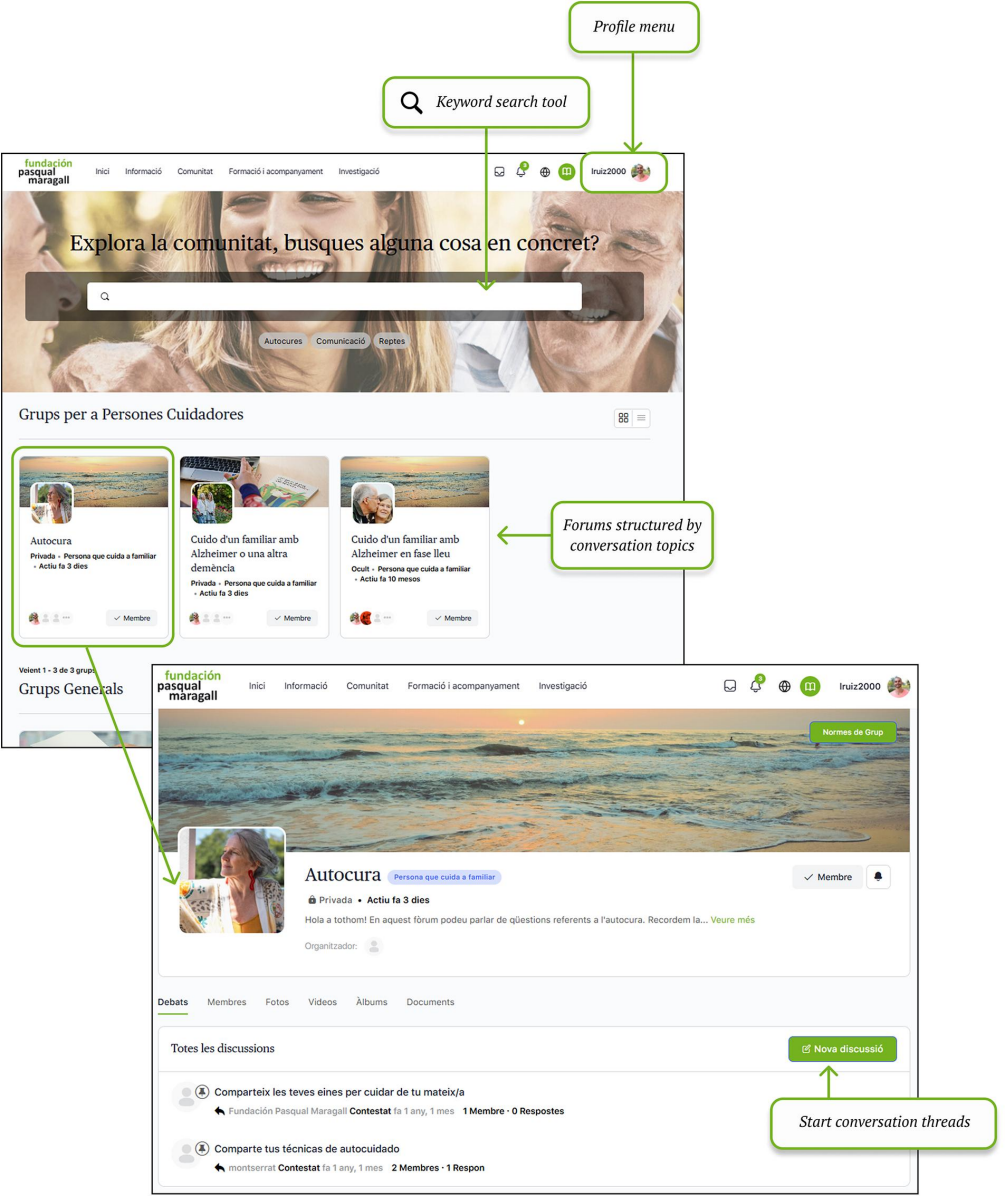
Community section: forums and conversation threads. Screenshot from: Pasquall Maragall Foundation, https://areaalzheimer.fpmaragall.org/.

Digital community channels the exchange of knowledge and experiences among people who share a connection with Alzheimer's disease. This section focuses on peer-to-peer connection (Figure 2).

**Figure 3:**
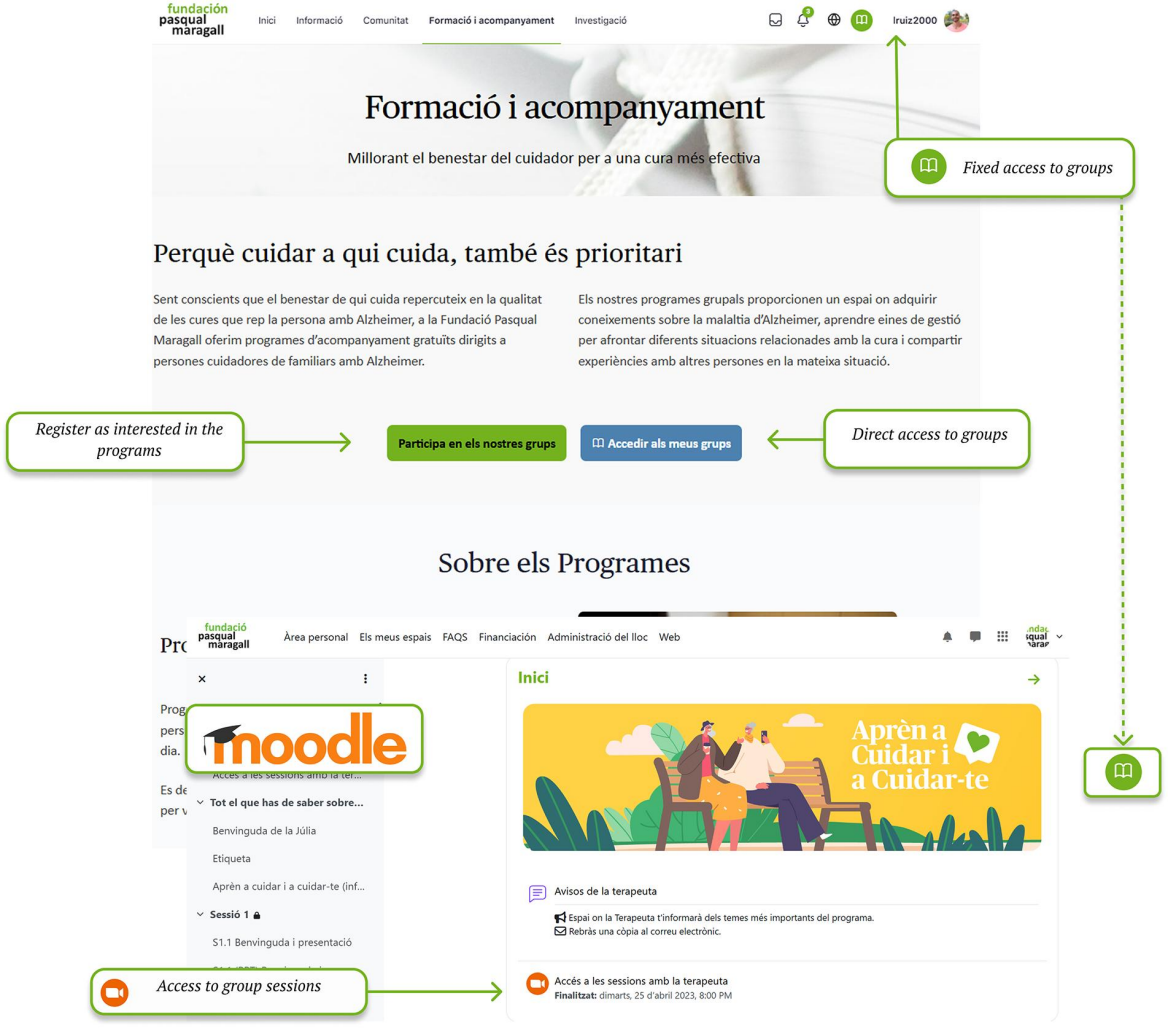
Training and support: registration and access to groups and moodle. Screenshot from: Pasquall Maragall Foundation, https://areaalzheimer.fpmaragall.org/.

Training and support programs offered by the Pasqual Maragall Foundation are held by professionals, providing access to a virtual classroom environment where they are carried out (Figure 3).

**Figure 4:**
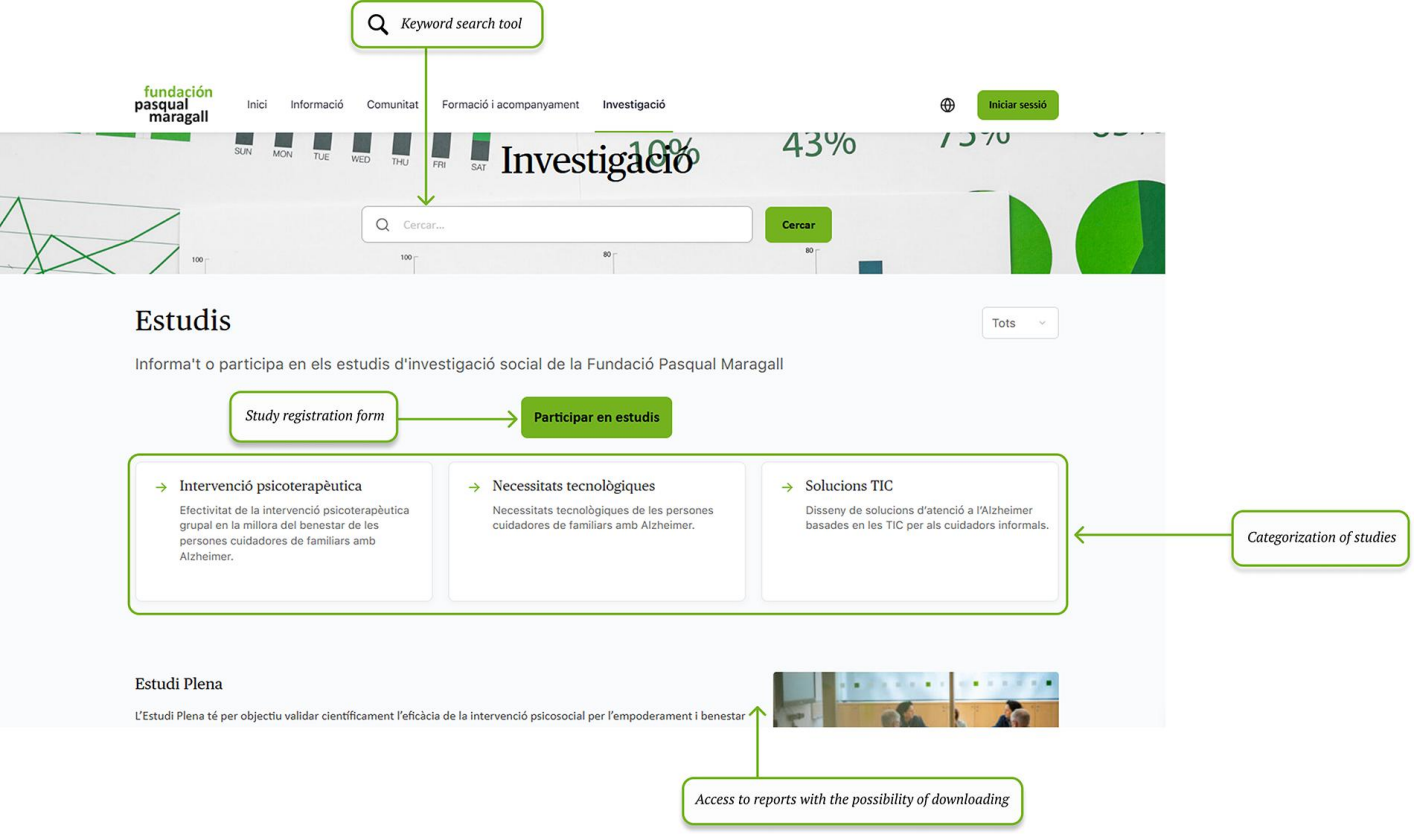
Investigation section: registration and access to studies. Screenshot from: Pasquall Maragall Foundation, https://areaalzheimer.fpmaragall.org/.

Investigation section contributes to generating specialized knowledge in the field of Alzheimer's disease care and makes this information available to users of the platform (Figure 4).

**Figure 5:**
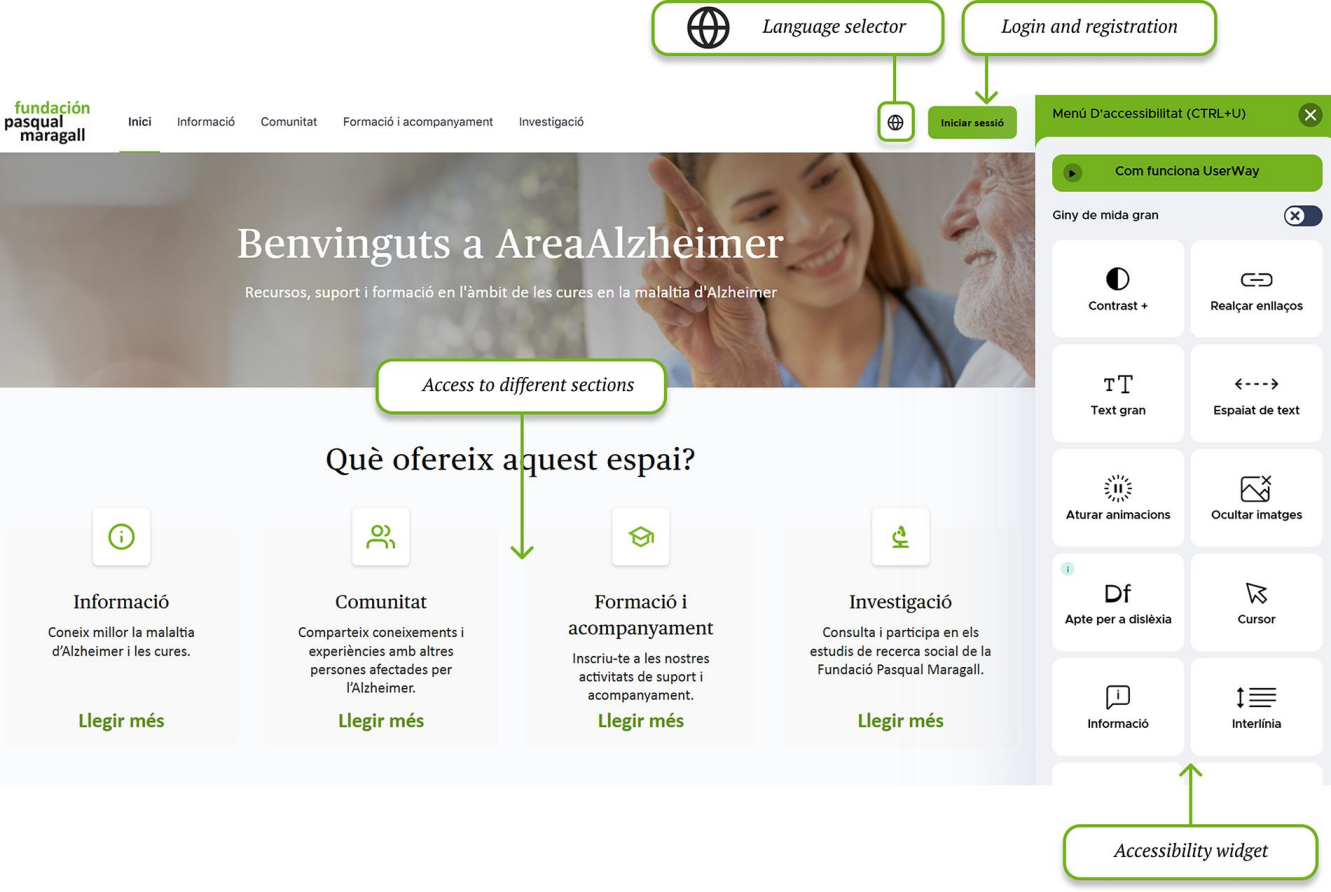
Home page section and generic tools. Screenshot from: Pasquall Maragall Foundation, https://areaalzheimer.fpmaragall.org/.

**Figure 6:**
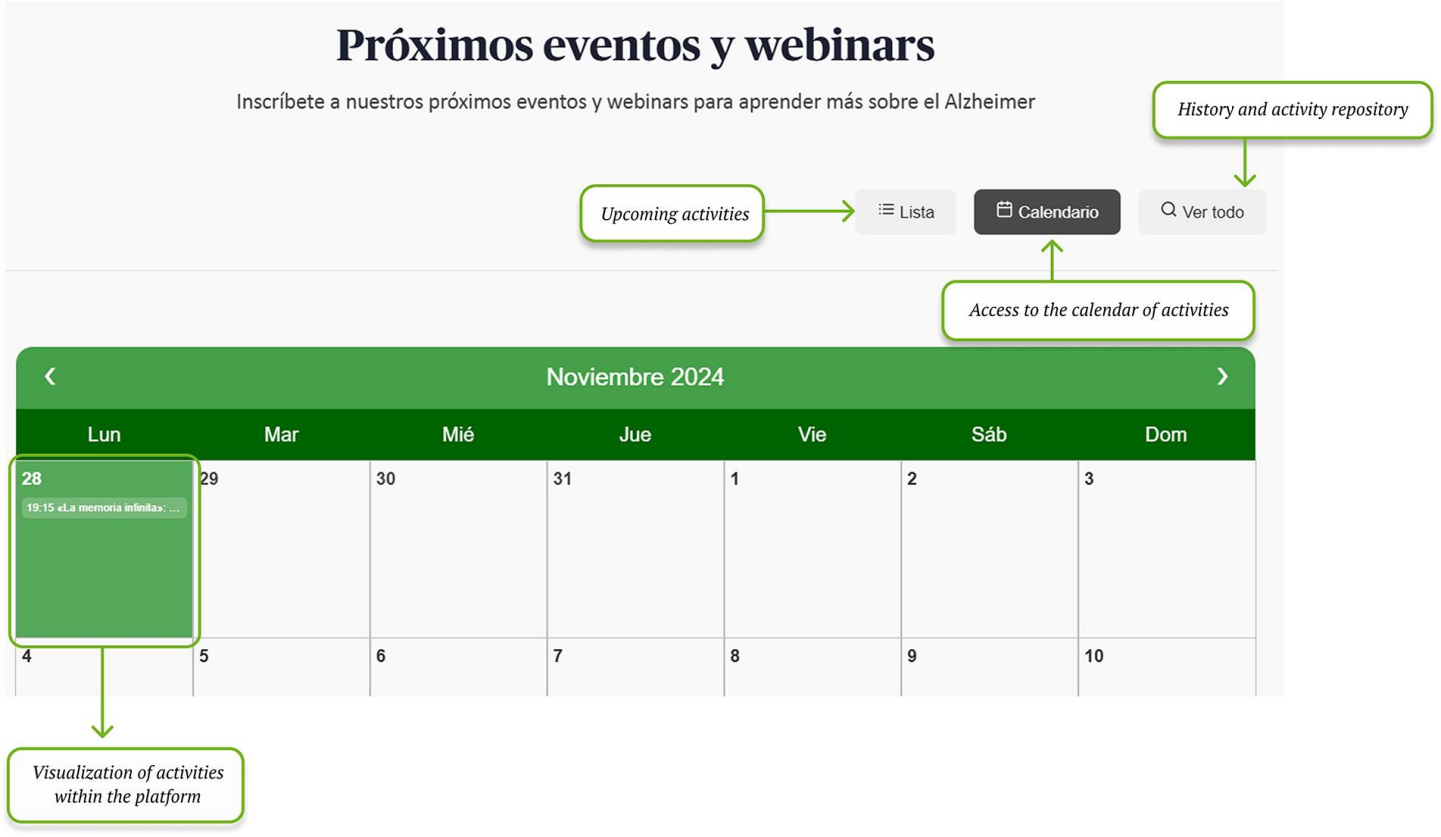
Event calendar: upcoming activities and repository. Screenshot from: Pasquall Maragall Foundation, https://areaalzheimer.fpmaragall.org/.

Through the home page, users can navigate to the different sections, while having access to other cross-cutting tools such as event calendar (Figure 5) and webinars (Figure 6) when published.

## 3 Material and methods

### 3.1 Research design

A mixed-methods approach was employed, integrating both quantitative and qualitative research techniques. This design enables the capture of not only objective and measurable data, but also the experiences and perceptions that cannot be fully understood through a purely quantitative lens. By combining both approaches, the study aims to provide a comprehensive and in-depth perspective on current training resources, the shortcomings identified, and the needs expressed by family caregivers.

From a quantitative standpoint, the research is based on the application of several surveys specifically designed for each phase of the project's development (24, 33–35).

In addition, focus groups and in-depth interviews were conducted to complement the information obtained through the surveys. The use of qualitative research techniques allows for a more detailed exploration of caregivers' experiences, perceptions, and needs. Such methods capture aspects that are not easily observable or measurable, such as emotions, ethical dilemmas, the experience of caregiver burden, or the subjective assessment of available resources.

**Figure 7:**
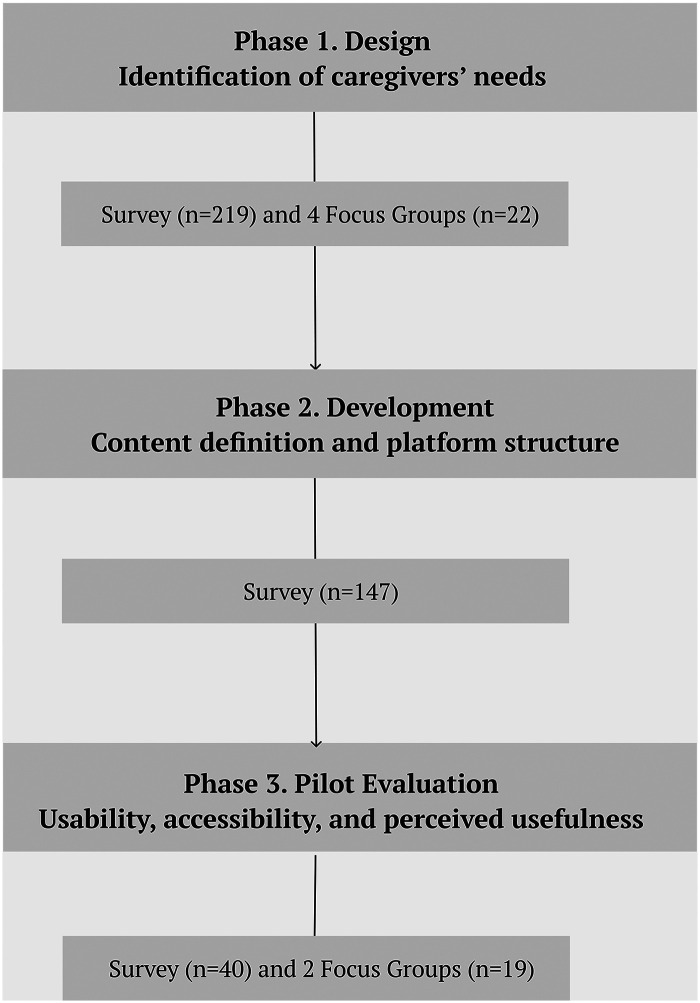
Development phases of the *AreaAlzheimer* digital platform.

The flow chart (Figure 7) summarises this structure, showing the progression of the research from the identification of needs to the evaluation of the prototype, together with the main techniques and instruments used at each stage

### 3.2 Development phases

The development of this project began in June 2022 and is currently in the evaluation and improvement stage. The design of *AreaAlzheimer* has been structured into three phases:

#### 3.2.1 Phase 1. design

The first phase focused on identifying and analysing the needs of family caregivers of individuals diagnosed with Alzheimer's disease. This information would then inform the functional and conceptual design of the future digital platform.

During this phase, a structured survey of 27 items was administered to 210 caregivers who met the inclusion criteria. The items were organised into previously validated dimensions (8) and incorporated new categories related to information about the disease and the use of technology (24).

In this phase of the study, the questionnaire was organized into different sections:

1. Sociodemographic profile: Questions to collect basic information about the respondents, and specific questions on the caregiver role regarding kinship and cohabitation.
2. Understanding current needs: Score of a selection of needs formulated from the literature classification (8) where some tasks and need categories were specified to facilitate and complete the self-administered online survey. Respondents scored them from 1 (unimportant) to 5 (very important).
3. Use of technology: This section of the survey explores participants' use of technological devices for health and caregiving purposes. It covers mobile phones, computers, and other devices, distinguishing between personal health use and caregiving tasks, with both closed and open-ended questions to capture frequency, purposes, and specific functionalities.
4. Perceived usefulness of available technology: This section explores perceived usefulness from technology surveyed caregivers use, and the extent to which these tools support daily care tasks, personal health, social well-being, financial and organizational challenges, and access to knowledge and services.

In addition, four focus groups (*n* = 22) were conducted to explore caregivers' perceptions, experiences, and technological barriers in depth. The groups were differentiated by age and relationship to the person being cared for (spouses, children, or siblings), with average ages ranging from 56 to 75 years.

Analysis of this phase allowed us to establish the main areas of need: (1) caregiving and organisational tasks, (2) personal health, (3) access to knowledge and professional support, and (4) balancing responsibilities. These results formed the empirical basis for defining the content and functionalities of the *AreaAlzheimer* platform.

#### 3.2.2 Phase 2: development

In the second phase, the focus shifted to validating caregiver's interest and preferences regarding potential platform materials. To this end, a self-administered online survey was conducted with the participation of 147 family caregivers of people diagnosed with Alzheimer's disease.

The questionnaire was structured in four sections:

1. Sociodemographic and care profile, including duration and type of relationship with the person being cared for.
2. Technological profile, with indicators of digital literacy and frequency of device use.
3. Content evaluation, where participants assessed the usefulness of different types of digital resources according to four previously identified areas of need.
4. Preferred access format evaluated using a 1–5-point Likert scale.

To measure digital literacy, a scale based on the European DigComp framework (36) was used, adapted to the characteristics of the study (Table 1).

**Table 1:** Digital literacy assessment levels.

Levels	Digital literacy
Lowest level	I perform tasks and navigate digital environments when receiving direct help from someone else.
Basic level	With appropriate guidance, I can perform autonomously simple tasks and navigate digital environments.
Intermediate level	Without help and independently, I can perform tasks, navigate and communicate in digital environments.
Advanced level	I can guide others to perform tasks, navigate and communicate in digital environments.

Adapted from Carretero Gomez et al. (36).

The results obtained in this phase guided the development of the platform's content and functionalities, prioritising practical resources (information about the disease, task management and emotional support) over technical or specialised content.

#### 3.2.3 Phase 3: pilot platform evaluation

The third phase of the study aimed to validate the technical functionality and user experience of the *AreaAlzheimer* prototype, to verify its usability, accessibility and perceived usefulness among family caregivers of people with Alzheimer's disease (35).

Building on the results of the co-creation process, the platform's initial design was structured around four main sections, each addressing key areas of user interests and needs.

- Information section provides Alzheimer's-related resources and materials according to users' main characteristics -such as stage of the disease and family relationship- so that the content remains relevant, personalized and on time. This structure aims to ensure that the information provided aligns with the users' current needs, avoiding materials that might arrive “too late” in the caregiving process.
- Digital community fosters peer connection and knowledge exchange among individuals who share personal or familial links to Alzheimer's disease. Responding to the needs identified in the initial assessment—particularly the sense of loneliness and the desire to connect with others who have lived similar experiences- the community serves as a safe space for emotional support, guidance, and shared learning.
- Training and support area provides family caregivers with access to professional guidance through structured programmes within a virtual learning environment (Moodle), which allows participation in group sessions and ongoing support throughout the caregiving journey,
- Investigation section addresses caregiver's interest in accessing current scientific knowledge and ongoing research. It includes a repository of studies and projects conducted by Pasqual Maragall Foundation, offers summaries of completed activities and results, and provides a registration form for users interested in participating in upcoming research activities.

Once the platform's sections and design were defined, a beta version was developed for pilot testing. The pilot evaluation was conducted in two parts: the first was an online evaluation. In this evaluation, participants used the platform. They also completed an evaluation survey. The second part involved a face-to-face test. In both parts, participants were asked to complete a series of tasks, including browsing the information section, participating in community forums, registering for support programmes, adjusting user settings and completing the same user experience evaluation survey.

The survey was divided into two sections:

1. Respondent profile: The first section asked about the basic sociodemographic characteristics of the family caregivers surveyed.
2. Evaluation of the *AreaAlzheimer* platform: In this second section, various questions were asked related to the three dimensions used to evaluate the tool: accessibility, usability, and perceived usefulness (25–27, 37, 38).

A total of 40 caregivers took part in this stage of the research, which was split into two groups: 21 completed the online evaluation and 19 participated in a face-to-face session at the Pasqual Maragall Foundation's facilities. During the face-to-face test, participants performed a series of standardised tasks, such as browsing sections, participating in forums or modifying profile settings, while their times and success rates were recorded.

The following validated scales were used to evaluate the platform. Table 2 shows he explanation of each scale.

- The System Usability Scale (SUS) was used to measure overall usability.
- The Single Ease Question (SEQ) assessed accessibility by task.
- The Perceived Usefulness Scale (PUS) and the Behavioural Intention Scale (BIS) were used to estimate perceived usefulness and intention to use, respectively.

During the test, participants interacted with the platform by completing a series of predefined tasks designed to assess their ability to navigate, understand the structure, and effectively perform basic actions within the digital environment. The tasks corresponded to the main sections of AreaAlzheimer. Table 3 describe the different tasks demanded.

**Table 2:** Dimensions and scales used to assess AreaAlzheimer platform.

UX Dimension	Description	Scale	Measurement
Usability	Measurement of the perceived ease of use of the platform through the level of agreement/disagreement with a series of items	System Usability Scale (SUS)	Likert scale from 1 to 5: (1) Strongly disagree and (5) Strongly agree
Accessibility	Subjective opinion of users on their experience of using the platform	Single Ease Question (SEQ)	Likert scale from 1 to 10: (1) very easy and (10) very difficult.
Usefulness	Individuals’ perceptions of how technologies can improve their tasks or functions.	Perceived Usefulness Scale (PUS)	Likert scale from 1 to 5:(1) Not at all (5) a great deal
	Perception of the intentions/behaviour of the users to start using a web-based intervention.	Behavioural Intention Scale (BIS)	Likert scale from 1 to 5:(1) Not at all (5) a great deal

**Table 3:** Description of tasks.

Space	Task
Information	• Browse the information section • Search and find articles that match their interest
Community	• Participate in the forum • Change the username
Training and support	• Search information about the intervention programs • Complete the form
Research	• Access profile settings. • Update the disease stage (if any changes have occurred).

Participants had a maximum of five minutes per block of tasks. During the face-to-face session, performance was recorded using screen recordings and direct observations by researchers, always ensuring the anonymity and confidentiality of the data.

Performance was evaluated using two main quantitative parameters:

- Efficiency: the user's ability to complete each task. A score of *1* was assigned if the task was completed correctly, *0.5* if it was partially completed, and *0* if it was not completed. The overall success rate was calculated using the following formula:
  $$\begin{array}{c} \mathrm{Success}\;\mathrm{rate}=(\mathrm{socre}\;\mathrm{obtained}\;\mathrm{in}\;\mathrm{performing}\;)x100\\ \mathrm{tasks}/\mathrm{maximum}\;\mathrm{possible}\;\mathrm{score} \end{array}$$
- Efficiency: measurement of the time spent on each task, expressed in minutes. Average values were calculated for the duration per task and thematic block.

These measurements made it possible to identify the most intuitive sections of the system and those that required simplification or redesign.

Finally, discussion groups took place after the evaluation of task performance. The aim of these groups was to gather additional impressions regarding participants' interaction with the platform. This allowed us to gain a deeper understanding of participants' experiences and identify areas for improvement related to the platform's usability.

The three methodological phases demonstrate a participatory process, where caregivers' perceptions informed each stage of development. The use of mixed methods and data triangulation strengthened the internal validity of the study and enabled the construction of a digital platform, tailored to the real needs of users.

### 3.3 Participant selection

Participants were recruited from the database of family caregivers maintained by the Pasqual Maragall Foundation. A purposive sampling strategy was applied, based on the following criteria: (1) being over 18 years of age, (2) caring for a relative diagnosed with Alzheimer's disease, and (3) having participated in at least one caregiver intervention program offered by the Foundation.

The Pasqual Maragall Foundation's database includes a total of 3.189 users who met the inclusion criteria, who were contacted by email during the different phases of the study to inform them about the project and invite them to take part in the development of the *AreaAlzheimer* platform. In total, 419 family caregivers of a person diagnosed with Alzheimer's disease participated in the various stages of the research.

### 3.4 Data analysis

Across the three phases of the study, a descriptive analysis of the survey data was carried out using SPSS 23. Frequency and percentages were calculated for sociodemographic and caregiving variables and means, and standard deviations were computed for Likert-type items and scales. This descriptive analysis allowed us to characterise the profile of the participating family caregivers, their perceived needs, their level of digital competence, their evaluation of different technological resources, and their experience of use and usability of the AreaAlzheimer platform throughout the different phases of the project.

Qualitative data were generated through focus groups conducted with family caregivers of people with Alzheimer's disease. In Phase 1, four focus groups (*n* = 22) explored caregivers' experiences, perceived needs, and technological barriers in depth, providing the basis for identifying key support domains and informing the initial design of the AreaAlzheimer platform. In Phase 3, qualitative data analysis focused on two on-site discussion groups, with 19 participants taking part. The qualitative phase included a usability pilot and a subsequent focus group discussion. During the usability pilot, participants were asked to complete a series of predefined tasks on the platform (described in Phase 3). The sessions were documented through screen recordings, which were later used to evaluate whether each task was completed and the time required to do so. In addition, five members of the research team acted as participant observers. They accompanied the sessions, provided support on how to navigate specific sections of the website without indicating how to perform the tasks, and took detailed notes on the main usability difficulties encountered by participants. In the second part of the session, a face-to-face focus group was conducted in which participants discussed their overall impressions of the platform and suggested improvements in terms of accessibility and usability. With participants' prior informed consent, the group discussions were audio-recorded and transcribed verbatim. All transcripts and screen recordings were anonymised before analysis by removing any identifying information.

Qualitative material from the focus groups was examined using content analysis supported by Atlas.ti 25. The analysis was carried out by three researchers, who independently read all transcripts and observation notes to become familiar with the material and obtain an overall understanding of caregivers' experiences and needs. An initial coding framework was then developed by combining deductive codes derived from the study aims and the discussion guide with inductive codes emerging from the data. This coding framework was systematically applied to the transcripts and refined in an iterative process. Any discrepancies in coding were discussed until consensus was reached. Finally, related codes were grouped into broader categories and themes that captured participants' perceptions of the platform, the main usability barriers, and suggestions for improving accessibility and user-friendliness.

The triangulation of quantitative and qualitative data strengthens the robustness of the analysis, enables validation of findings across different levels of reality, and provides a solid empirical foundation for the development of user-centred technological tools.

## 4 Results

### 4.1 Sociodemographic profile

The *AreaAlzheimer* platform was developed with the participation of 419 caregivers of family members diagnosed with Alzheimer's. Of the 387 caregivers who participated in the quantitative methods through surveys in each phase of the study, most were women (67.44%), with men representing 32.56% of the sample. The average age of the participants was 60, and most were daughters or sons of the family members they care for (52.71%), followed by spouses or partners (40.05%). In terms of living arrangements, most participants stated that they live with the family member they care for (65.89%). Table 4 shows sociodemographic results of the sample.

**Table 4:** Sociodemographic profile of the participants.

Edad	X	SD
Average age	60.03	0.47
Sex	*n*	%
Male	126	32.56
Female	261	67,44
Total	387	100
Kinship		
Son/Daughter	204	52.71
Spouse/Partner	155	40.05
Brother/Sister	5	1.29
Nephew/Niece	6	1.55
Son/Daughter in law	2	0.52
Other relatives	14	3.62
We are not related	1	0.26
Total	387	100
Living together		
No	132	34.11
Yes	255	65.89
Total	387	100

### 4.2 The technological profile and use of technology in meeting the needs of caregivers

The first two phases of the research aimed to gain a detailed understanding of the participants' technological profiles and how they use technology to address various concerns or needs arising from their role as caregivers.

#### 4.2.1 Phase 1: design of areaAlzheimer platform

The first phase of the research analyses the use of technology and the needs of caregivers, with the aim of understanding how technology can support and enhance their lives.

**Figure 8:**
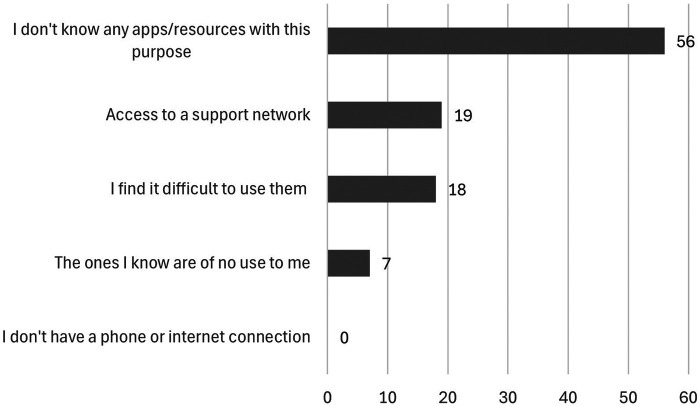
Reasons why they do not use their mobile phones to search for information to make decisions as caregivers.

Results showed that 65% of caregivers use their mobile phones to obtain information and make decisions regarding care. Among those who do not use mobile phones (35%) or do not find them useful (14%), the main reason is a lack of knowledge about technological resources with this purpose (56%), followed by a lack of support network to help them to use this apps and difficulties in use (19%), or the perception that they are not useful (7%) (Figure 8).

#### 4.2.2 Phase 2. Development of AreaAlzheimer platform

**Figure 9:**
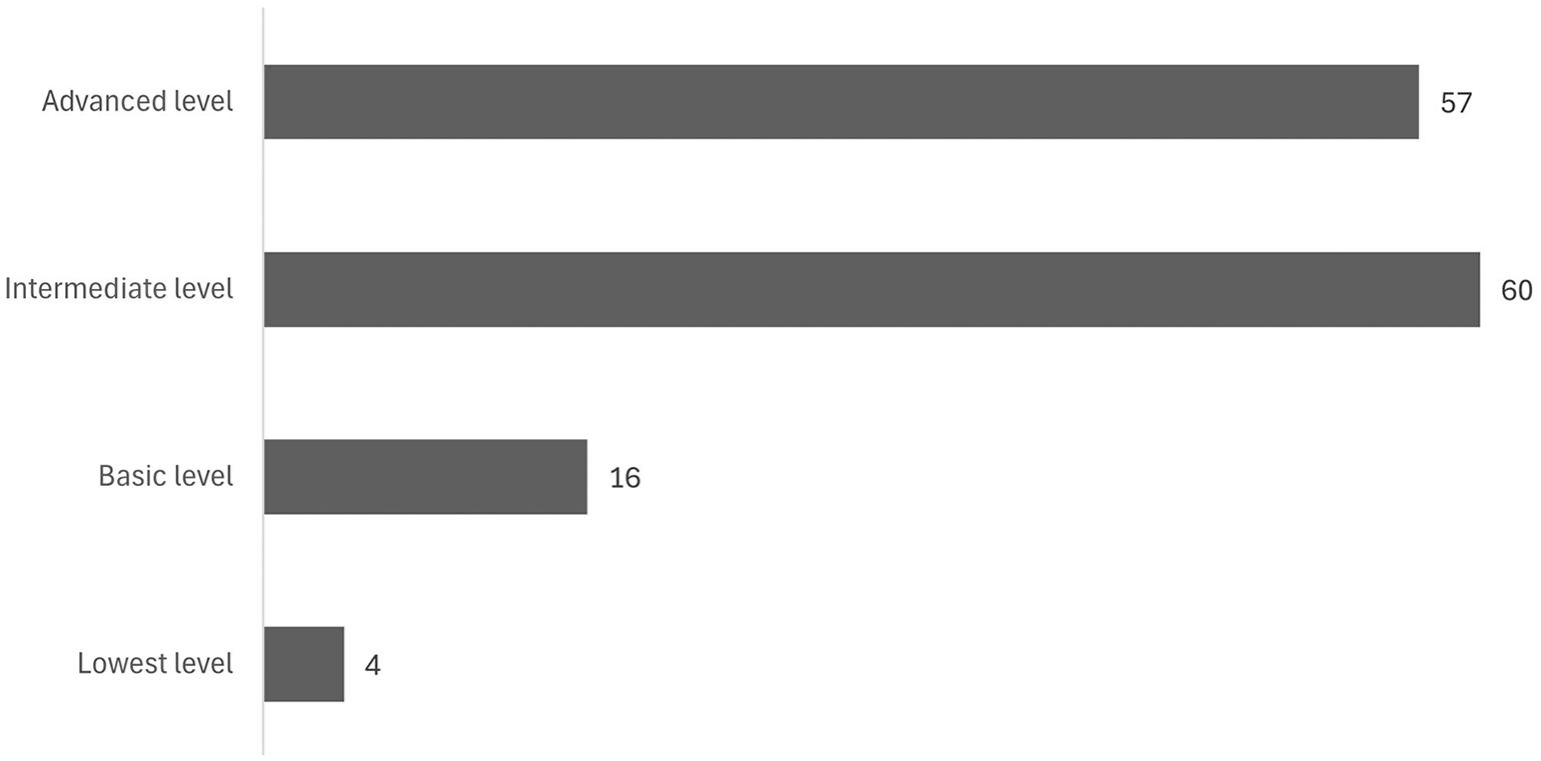
Self-assessment of technological skills based on Table 1 categories.

In the second phase of the research, participants were asked to self-assess their digital skills. Most rated themselves as having intermediate (43.8%) or advanced (41.6%) skills. The use of digital devices daily was confirmed by 63.2% of respondents, with smartphones (45.6%) and computers (44.1%) being the primary devices (Figure 9). The risks and limitations perceived in the use of technology were impersonality (22.6%) and concerns about privacy and security (21.9%).

Access to information was one of the most highly valued areas in the first phase, with information on Alzheimer's disease (75%) and future planning (67%) proving particularly popular. A similar pattern was observed in the second phase, where the highest-rated digital content in this area concerned general knowledge of the disease and its symptoms (M = 3.45; SD: 2.06: Range: 1–6), as well as searching for and requesting social resources (M = 3.26; SD: 1.4; Range: 1–6). In comparison, scientific information on medical advances received lower usefulness ratings (M = 2.43; SD:1.5; Range: 1–6). Taken together, these descriptive differences may indicate that, within our sample, caregivers tended to rate more highly content that was directly applicable to everyday care than more specialised or technical information, although this interpretation should be considered with caution given the descriptive nature of the analyses.

With regard to supervision and support for caregiving tasks, although participants highlighted task supervision (67%) and the need for help from others (66%) as priorities during the design phase, these demands are in line with the content identified as most relevant during the development phase, with a positive assessment of the development of digital tools for organising and planning care, as well as calendars for medical appointments and other tasks. These demands are in line with the content identified as most relevant in the development phase, with positive assessments of the development of digital tools for organising and planning care, as well as calendars for medical appointments and medication reminders (M:3.82; SD: 1.2; Range: 1–5). However, the usefulness attributed to other management resources, such as daily routine checklists (M:2.42; SD: 1,6; Range: 1–5), was low, indicating that caregivers place greater value on technological applications that respond to tasks that are relevant and have an impact on the well-being of the person being cared for.

**Figure 10:**
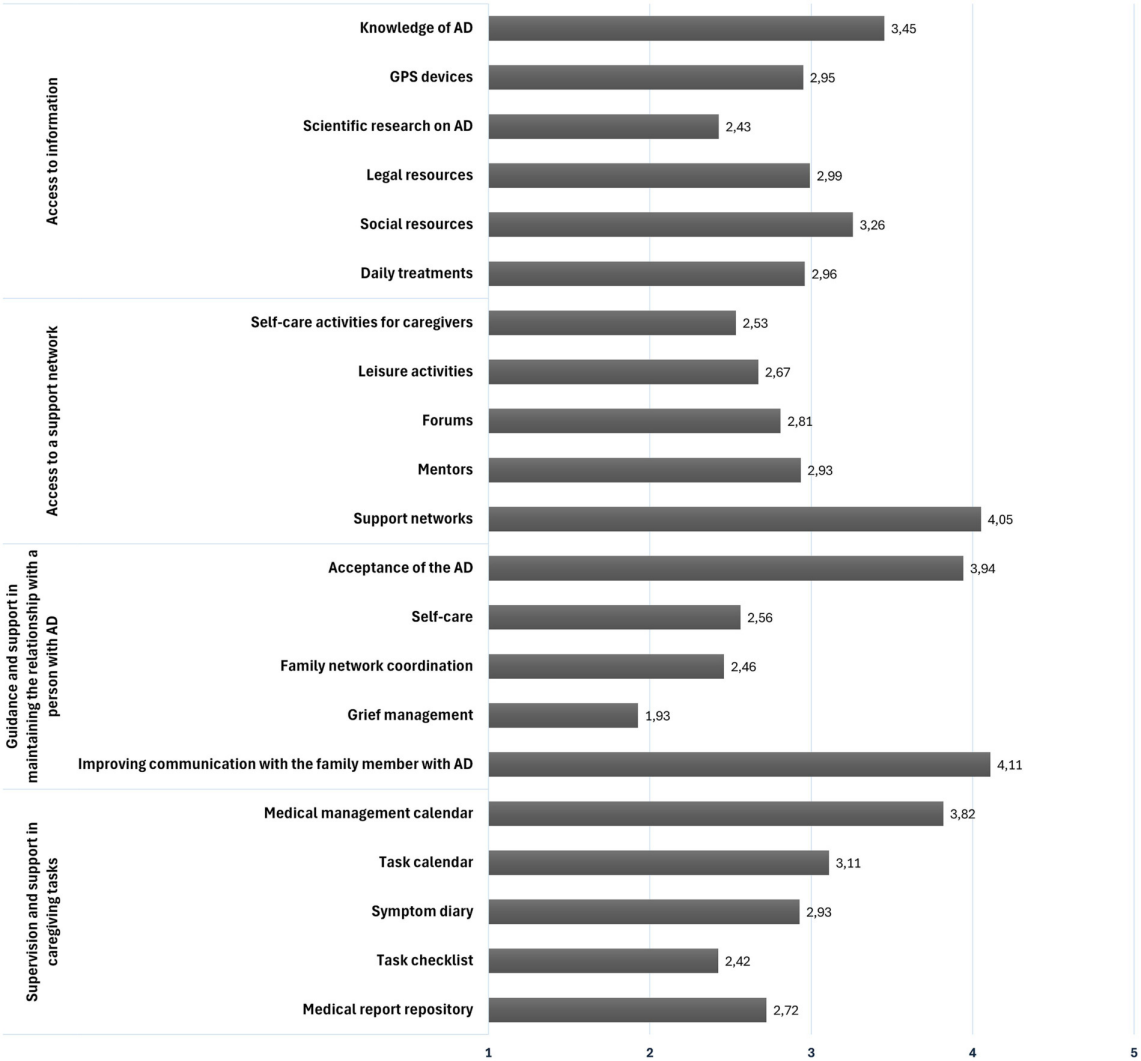
Most highly rated content in the development of the *AreAreaAlzheimer* digital platform.

In terms of the area of need related to guidance and support in relationships with people with Alzheimer's, related to the dimension of personal health, the results of the first phase reflect that caregivers prioritised psychological health (59%) over physical health (45%). This need is reflected in the choice of digital content related to professional support evaluated in the second phase. There, resources related to improving communication with the person being cared for (M: 4.11; SD: 0.99; Range: 1–5) and acceptance and understanding of the disease (M: 3.94; SD: 1.29; Range: 1–5) received high ratings. These aspects both contribute to emotional coping and reducing psychological overload, providing a meeting point between the detected needs and the responses that digital resources can offer. Figure 10 shows the most highly valued content in the design phase according to each of the areas of need identified in the first phase of the research.

In addition, the results obtained from the analysis of the focus groups carried out during the development phase highlighted the importance of having accurate, personalised information tailored to the different stages of the disease and their daily reality.

“The problem is that we don't know what it will be, because it's the first time we’re facing this situation. So for me… my need is to know that there is someone who has gone through caring for someone with the disease, and is a real person, a place I can turn to, even if it's online, as you say (addressing the moderator), and where I can ask, “Hey, this is happening to me, what do I do?” Or that, this person has certain topics to go through and can say, “Look, in such-and-such stages you’ll start to experience this.” Right? “ (FG2_Q1)

“Each person is affected in a different way even when they’re at the same stage. So everyone has their own needs, especially when it comes to treatments.” (FG4_Q3)

“That there were a specific professionals assigned to the Foundation (…) so that even if it was just one day a month or once every fifteen days, we could have contact with them.” (FG4_Q1)

They asked for recommendations for monitoring devices, and advice on legal questions (FG1_G1). They also focused on the peer-to-peer emotional support needed as caregivers.

“Advising you on devices, advising you on many different things: everything to do with recommendations about geolocators, legal advice..” (FG1_Q1).

“To talk with people who are more or less in the same situation, because it's very different to talk about this disease with people who know what it's about.” (FG4_Q2).

“Like a forum, you know? Where people answer each other.”(FG3_Q6).

“Once I will be no longer here (in this group) I will feel alone in caring for my father. That's what I see as… or what scares me the most: being faced with a situation where I don't know what to do. Being able to have someone to turn.” (FG2_Q1).

### 4.3 Pilot evaluation of the *areaAlzheimer* digital platform

A pilot version of the AreaAlzheimer platform was developed based on the results obtained in the first two phases. Table 5 shows the average scores obtained on each scale.

**Table 5:** Average scores obtained for accessibility of AreaAlzheimer.

Accessibility (single ease question)	Mean value (SD) (*n* = 40)
Navigate to the section where information can be found	3.92 (2.85)
Search and find articles that fit your interests	4.3 (2.72)
Participate in the forum	5.38 (3.24)
Modify your username	4.29 (3.33)
Find information about the programmes	4.05 (2.63)
Access profile settings	3.89 (3.10)
Modify the phase of the disease (if there have been changes)	5.57 (3.24)
Total Score	4.58 (3.02)

Accessibility received one of the highest scores, with an average of 4.6 out of 10 (SD: 3.02; Range:1- 10). However, analysis by task revealed important nuances. Activities such as accessing the profile (M:3.89; SD: 3.10; Range:1–9) and searching for programme information (X: 4.05; SD:2.63; Range:1–9) were considered easy, whereas participating in the forum (M: 5.38; SD: 3,24; Range:1–10) and updating the stage of the disease (M:5.57; SD: 3.24; Range:1–10) were perceived as more challenging. This may indicate that, although the platform is generally accessible, some specific functionalities require simplification.

However, during the face-to-face session, it was identified that the most difficult tasks were related to accessing profile settings and changing the username, with success rates of 21.15 and 30.77 points respectively. These tasks also took the least time to complete. These tasks were the ones that fewer people managed to complete, as is also confirmed when checking the time taken to complete each one. In this case, only seven people managed to successfully change their username, while ten people accessed their profile settings. As these two tasks are related, this means that three of the ten people who successfully accessed their profile were ultimately unable to change their username on the platform.

In contrast, the highest success rates were achieved in tasks such as browsing the information section (65.30 points) and searching (63.43 points), followed by updating the stage of the disease (53.85 points) and searching for and sharing articles of interest. The average time taken to complete these tasks was six minutes. Table 6 shows the success rate for each task and the average time taken to complete it.

**Table 6:** Task performance.

Task description	Effectiveness (*n* = 19)	Efficiency (*n* = 19)
Browse the information section	65.38	0:03:53
Search for and share articles of your interest	53.85	0:09:34
Participate in the forum	42.31	0:06:17
Change your username	30.77	0:01:47
Search for information about intervention programmes	36.54	0:04:38
Complete register form	32.69	0:03:16
Access your profile settingas	21.15	0:02:15
Update the disease stage	63.46	0:06:37

On the other hand, usefulness received the lowest score of the three dimensions (M:3.4; SD: 1.08; Range:1–5). While caregivers rated aspects such as clarity (M: 3.36; SD: 1.14; Range:1–5) and ease of use (M: 3.41; SD: 1.05; Range:1–5) positively, they also noted that the current content and functionalities do not fully meet their needs. This result is linked to the pilot nature of the platform, which does not yet offer the full range of services planned.

Meanwhile, the intention to use the platform scored very high (M: 4.5; SD: 0.75; Range: 1–5), reflecting a strong willingness to use the platform in the future. The main motivations highlighted were access to information about the disease (M: 4.60; SD: 0.63; Range: 1–5), participation in programmes for caregivers (M: 4.58; SD: 0.67; Range: 1–5), and recommending the tool to other caregivers (M: 4.55; SD: 1.11; Range: 1–5). This difference between high intention to use and lower perceived usefulness suggests that caregivers see great potential in *AreaAlzheimer* but expect improvements in content and functionality.

Finally, the overall usability score (SUS) was 74.3 out of 100, which is above the standard threshold of 68 indicating acceptable usability. The highest-rated items were desire for frequent use (M: 4.28; SD: 1.12; Range:1–5), ease of use (M: 4.10; SD: 0.97; Range: 2–5), and content adequacy (M: 4.10; SD: 0.79; Range:2–5). However, the perception that “most people would learn to use it quickly” scored an average of 3.13 (SD: 1.14; Range:1–5), as did confidence in using it, showing room for improvement, especially regarding the learning curve for new users. Table 7 shows the results obtained in the other scales applied.

**Table 7:** Average scores obtained in the AreaAlzheimer assessment.

Perceived usefulness scale	Mean (SD) (*n* = 40)
Platform is easy to learn and use	3.33 (1.05)
AreaAlzheimer Platform is clear and understandable	3.36 (1.14)
AreaAlzheimer Platform is easy to use	3.41 (1.04)
Total Score	3.36 (1.08)
Behavioural Intention Scale	
How much would you like to use AreaAlzheimer?	4.50 (0.75)
Would you recommend AreaAlzheimer to other caregivers you know?	4.55 (0.60)
Would you use the AreaAlzheimer to get access to the Foundation's programmes for caregivers?	4.58 (0.67)
Would you use AreaAlzheimer to find out more about the disease?	4.60 (0.63)
Would you use AreaAlzheimer to get in touch with other caregivers?	4.13 (1.11)
Total Score	4.47 (0.75)
System Usability Scale	
I thought I would like to use the AreaAlzheimer platform frequently.	4.28 (1.12)
I have found access to the AreaAlzheimer platform too difficult.	1.97 (1.11)
I thought that using AreaAlzheimer was easy for me.	4.10 (0.97)
I thought I would need the support of a technician to be able to use AreaAlzheimer.	1.78 (1.29)
I thought that the selection of content was adequate	4.10 (0.79)
I thought that there are many errors in the contents of the AreaAlzheimer platform.	1.71 (1.02)
I thought that most people would learn to use AreaAlzheimer quickly.	3.13 (1.14)
I thought that AreaAlzheimer was too complicated to use.	2.26 (1.14)
I felt very confident using AreaAlzheimer	3.77 (1.01)
I think I need to learn a lot of things before I start using AreaAlzheimer	1.85 (1.19)
Total Score	2.89 (1.08)

Finally, the focus groups provided important insights that helped us to understand the significance of the survey evaluation scores and identify areas for improvement.

In terms of accessibility, the high overall score was supported by comments on the visual design, which participants described as “fresh and attractive” because of the use of light colours and images (FG1_Q2). This coincides with favorable scores for tasks such as accessing the profile (M: 3.89) and searching for information about programs (M: 4.05), both of which were considered easy. However, the difficulty noted in tasks such as participating in the forum (M: 5.38) or changing the stage of the disease (M: 5.57) was also commented on in the focus groups, where some caregivers pointed out that certain sections were less intuitive, especially for people with less digital experience (FG1_Q4).

“(..) When I accessed it, I was struck by the use of very light colours and lots of images, which made it look fresh and visually appealing” (FG1_Q2).

“The content is very interesting to us, but it needs to be more straightforward and easier to navigate. Also, we are mentally accustomed to a specific type of website, where you click and drop-down menus appear, or you click on an image and it opens. So, in a way, you expect that kind of experience.” (FG1_Q4)

Regarding perceived usefulness, the moderate score of 3.4/5 was reflected in the qualitative comments. Participants positively rated the presentation of information, in particular short formats with an indication of reading time (FG1_Q1), which is in line with the relatively high scores for clarity (M: 3.36) and ease of use (M: 3.41). However, they explained that the actual usefulness would be enhanced if the platform offered more practical and personalised content, such as downloadable materials, action guides and activities to do with the person being cared for (FG1_Q5). These observations explain why, despite the perceived ease of use, the usefulness rating is lower.

“Yes, the content looks good, the images are appealing, and it grabs your attention, it keeps you engaged (…). I find the idea of 10-minute readings or 4-minute readings interesting because, you know, if you're on the metro or the bus, I think, “Oh look, 4 min, I have time,” and I read it. It's great that it displays the reading time—I found that to be a positive aspect (..)” (FG1_Q1).

“Perhaps a section in the menu or an environment where downloadable tools, worksheets, or memory workshops could be available? (…) Even if it's just exercises, so that they can have activities at home (…) maybe basic guidelines for things they can do or ways to assist at home. I think that would be great.” (FG1_Q5)

In terms of the intention to use, the high score of 4.5/5 reflects expressions of enthusiasm for features such as access to programs for caregivers, obtaining information about the disease, and interacting with other caregivers. The positive comments regarding interest in future service offerings explain why this dimension remains high, even though the perceived usefulness is lower at present (FG2_Q2).

“I found the content excellent. Once it becomes available, I will definitely use it because the forum and everything related to it seem like a great meeting point (..).” (FG2_Q2).

The usability of the platform was satisfactory, with an overall score of 74.3/100, but some aspects received lower ratings. These included the perception that most people would learn to use it quickly (M: 3.13) and confidence in using the platform (M: 3.77). This coincides with qualitative observations on the need to improve intuitive navigation. Suggestions such as highlighting titles when the cursor hovers over them, increasing text size or adding icons to guide reading seek to precisely reinforce confidence and reduce the learning curve (FG2_Q3). Comments on adapting to different caregiver profiles, especially older caregivers or those with low digital literacy, reinforce this point by explaining some of the differences in perceived ease of use (FG1_Q3).

“I was suggesting that, in some way, when you hover the mouse over a title, it should be highlighted or enlarged. The text is very small, very cramped, and hard to read. Also, maybe an icon or something that would help to understand the reading better.” (FG2_Q3)

“It is true that if you're targeting an audience more focused on caregivers, we are probably not the typical users. I mean, there are older people and younger people, but we all are users of this. A person who is not accustomed to a standard website may find it a bit more challenging. However, for the rest of the audience, I believe it's quite standard.” (FG1_Q3)

## 5 Discussion

The AreaAlzheimer project offers a case study on the implementation and challenges of technology-based interventions for informal caregivers of people with Alzheimer's disease in Catalonia. Building on the quantitative and qualitative results, the discussion connects these findings to existing literature and outlines implications for future digital tools for caregiver support.

One of the first elements to note is the explicit alignment between the project's sample demographics and the widely documented caregiver profile in Spain and similar contexts: most caregivers are women (67.44%), mostly adult children and spouses, with a mean age of 60 years. This coincides with Spanish National Institute of Statistics (INE) demographic data and international findings that caring responsibilities, especially for dementia, disproportionately fall on middle-aged and older women—an aspect repeatedly flagged in the literature as both a social inequality and a determinant of the types of digital solutions needed (12, 39). The relatively high level of cohabitation among study participants (65.89% living with care recipients) further underscores the intensive nature of caregiving in the sample, reflecting the intense emotional and practical pressures that digital platforms must address.

Technological profiles reveal a heterogeneous picture. Most respondents report intermediate or advanced digital literacy according to the adapted DigComp framework, yet a significant minority—particularly caregivers over 65—fall within basic or non-autonomous usage levels. This echoes work highlighting that the digital divide is driven not only by access, but also by confidence, habitual use and specific skills (23, 28). That around one third of caregivers do not use mobile phones for caregiving tasks, mainly due to lack of knowledge or perceived usefulness, suggests that technological solutions must be accompanied by explicit strategies to foster digital inclusion.

The user-centred, iterative structure of AreaAlzheimer is consistent with recommendations from systematic reviews (7, 12, 26). Four main priority needs—updated and practical information, support in care tasks, emotional guidance and relationship management, and access to a support network—mirror the multidimensional challenges described in previous research and reinforce the importance of interventions that integrate practical, emotional and social components (8, 26, 40). Caregivers in this study clearly prioritised immediately usable content (symptom management, routines, legal and administrative information) over more academic or abstract information, supporting calls for highly tailored, context-sensitive digital resources (8, 25).

Existing digital platforms for dementia caregiving frequently target needs like those identified in AreaAlzheimer, including information provision, support for care tasks and emotional counselling. However, many tools have been criticised for generic, one-size-fits-all content, limited personalisation and modest engagement with interactive features (7, 28). In line with these observations, AreaAlzheimer confirms that caregivers prioritise practical, context-specific information on symptom management, service navigation and daily care organisation over abstract or purely biomedical content, and value opportunities for peer connection and structured programmes (8, 17, 27). At the same time, the platform extends previous work by integrating four components—information, community, training and research—within a single environment, addressing informational, emotional and social needs in a more holistic way than many existing tools (21, 27).

AreaAlzheimer therefore addresses a key innovation gap in the field by moving beyond single-focus, generic digital tools towards a participatory, context-specific platform that combines information, community, training and research functions within a single environment. Unlike many stand-alone resources, it is co-designed with caregivers, explicitly incorporates their needs, expectations and digital literacy profiles, and is anchored in the Catalan social and healthcare system with a view to future integration into routine services. In addition, by including a dedicated research component, AreaAlzheimer functions not only as a support tool but also as an infrastructure for generating knowledge about caregivers’ needs and vulnerabilities, thereby informing more effective and equitable dementia-care policies.

From a usability perspective, AreaAlzheimer's performance is comparable to, or slightly higher than, that reported by other web-based interventions. Global usability scores above commonly accepted thresholds (SUS = 74.3/100) and positive perceptions of visual design and ease of navigation align with recent feasibility and implementation studies (26, 27, 38). Difficulties in more complex tasks—such as modifying profiles or using forums—mirror recurrent challenges in other platforms, where advanced features are often underused or perceived as unintuitive (41, 42). These parallels suggest that adding functionalities alone is insufficient; interfaces must be simplified, aligned with users' mental models and explicitly adapted for those with low digital confidence (23, 28).

The findings also reinforce concerns about the digital divide in dementia caregiving. Despite widespread access to smartphones and computers, many caregivers face barriers related to limited skills, lack of confidence, distrust in technology or privacy concerns, particularly among older adults (23, 28, 41). Similar patterns have been described in evaluations of web-based and mobile interventions, where older or less digitally literate users are less likely to register, participate or sustain long-term use (12, 25). AreaAlzheimer fits within this landscape, highlighting that digital innovation must be accompanied by inclusive design strategies—simplified interfaces, clear tutorials and alternative access routes—to ensure equitable benefits and avoid exacerbating existing inequalities.

An additional key finding concerns the relationship between perceived usefulness and behavioural intention to use. In the pilot, usefulness received the lowest score of the three user-experience dimensions, while intention to use was very high. This suggests that, although current content and functionalities are still limited, caregivers perceive strong potential value and are willing to engage as the platform evolves. The gap between moderate perceived usefulness and high intention to use echoes broader evidence in digital health, where early adoption often depends on perceived potential and trust in the sponsoring institution (7, 27, 40). In this sense, AreaAlzheimer illustrates how a participatory, co-designed development process can foster engagement intentions while generating a user-driven roadmap for optimisation and scaling.

Overall, these dynamics highlight the importance of continuous participatory research and agile development in digital dementia care. Caregivers' demands for personalised, phase-adapted content, downloadable tools and interactive, community-based features should remain central to future iterations (21, 43). Beyond this specific platform, the results support broader recommendations that digital interventions for dementia caregiving should combine robust empirical needs assessment, user-centred and inclusive design, and iterative, mixed-method evaluation to maximise feasibility, equity and scalability.

### 5.1 Strengths, limitations and future research

This study has several strengths. It provides an in-depth, user-centred evaluation of a novel digital platform specifically designed to support family caregivers of people with Alzheimer's disease. The mixed-methods, iterative approach and the involvement of multiple stakeholder groups (caregivers and professionals) offered a nuanced understanding of needs, expectations and early use experiences, which is essential for the further development of AreaAlzheimer.

However, the study has limitations that should be considered. First, it was conducted in a single regional context (Catalonia, Spain) with a predominantly female, middle-aged sample of caregivers, which may limit the transferability of the findings to other settings. Second, the pilot evaluation focused on a prototype and short-term use, so long-term engagement and effects on caregivers' well-being could not be assessed. Third, participation required basic access to digital devices and willingness to respond online, which may underrepresent caregivers with very low digital literacy or without stable internet access.

Future work should therefore include larger and more diverse samples, as well as longitudinal designs that assess the impact of AreaAlzheimer on key outcomes such as caregiver burden, mental health, quality of life and care-related decision making. Experimental or quasi-experimental studies comparing AreaAlzheimer with usual care or with other digital interventions would help to clarify its added value and cost-effectiveness. It will also be important to refine and test specific strategies to support caregivers with low digital skills—such as simplified interfaces, tailored onboarding, assisted use via professionals or peers, and blended formats that combine digital and face-to-face support—and to explore complementary offline materials for those with limited internet access.

Finally, regarding long-term implementation, next steps will involve co-designing a more refined and potentially simplified version of the platform for users with lower digital literacy, and with cognitive impairment integrating AreaAlzheimer into routine health and social care pathways, and evaluating its use under real-world conditions. Pragmatic trials and implementation studies will be needed to understand how the platform can be sustainably embedded in existing services, how professionals can support its uptake, and how it can be scaled up to reach underserved populations. Future iterations will therefore explore how the contents, navigation and potentially even the overarching branding (e.g., a broader dementia focus) can be adapted to ensure relevance and acceptability across different dementia diagnoses and care contexts.

## 6 Conclusion

AreaAlzheimer was developed through a rigorous user-centred, participatory mixed-methods process, illustrating how co-design with family caregivers can guide the development of digital tools that genuinely address their priorities. The platform demonstrated good usability and accessibility, and caregivers expressed a strong intention to use it, particularly to obtain practical and stage-adapted information, participate in structured support programs, and connect with peers for emotional and social support. At the same time, moderate usefulness ratings, task-specific difficulties, and marked differences in digital competence—especially among older caregivers—highlight the need to refine navigation, simplify complex actions, expand personalised content, and systematically address digital inclusion. These findings reinforce the value of robust needs assessment, user-centred design, and ongoing iterative development grounded in caregivers lived experience. Overall, AreaAlzheimer provides a solid foundation for scalable, inclusive digital support with the potential to improve caregivers' day-to-day care, emotional well-being, and outcomes for families facing Alzheimer's disease, while pointing to clear priorities for further optimisation and wider implementation.

## Data Availability

The raw data supporting the conclusions of this article will be made available by the authors, without undue reservation.
